# A Systematic Review and Meta-Analysis on the Association between Inflammatory Bowel Disease Family History and Colorectal Cancer

**DOI:** 10.1155/2021/4874459

**Published:** 2021-10-23

**Authors:** Hadis Najafimehr, Hamid Asadzadeh Aghdaei, Mohamad Amin Pourhoseingholi, Hamid Mohaghegh Shalmani, Amir Vahedian-Azimi, Matthew Kroh, Mohammad Reza Zali, Amirhossein Sahebkar

**Affiliations:** ^1^Basic and Molecular Epidemiology of Gastrointestinal Disorders Research Center, Research Institute for Gastroenterology and Liver Diseases, Shahid Beheshti University of Medical Sciences, Tehran, Iran; ^2^Gastroenterology and Liver Diseases Research Center, Research Institute for Gastroenterology and Liver Diseases, Shahid Beheshti University of Medical Sciences, Tehran, Iran; ^3^Student Research Committee, Hamadan University of Medical Sciences, Hamadan, Iran; ^4^Digestive Disease and Surgery Institute, Cleveland Clinic Lerner College of Medicine, Cleveland, OH, USA; ^5^Biotechnology Research Center, Pharmaceutical Technology Institute, Mashhad University of Medical Sciences, Mashhad, Iran; ^6^Applied Biomedical Research Center, Mashhad University of Medical Sciences, Mashhad, Iran; ^7^School of Pharmacy, Mashhad University of Medical Sciences, Mashhad, Iran

## Abstract

**Background:**

Colorectal cancer (CRC) and inflammatory bowel disease (IBD) are closely interrelated. However, the effect of having a family history of one disease on the risk of another remains undetermined.

**Aim:**

The purpose of this meta-analysis was to estimate the prevalence of a family history of CRC among patients with IBD, as well as the prevalence of a family history of IBD among patients with CRC.

**Methods:**

PubMed, Scopus, Embase, Web of Science, and Google Scholar were searched to identify studies reporting the prevalence of family history of IBD among patients with CRC, in addition to the prevalence of family history of CRC among IBD patients. Criteria for study inclusion consisted of the following: (1) studies that evaluated either IBD or CRC and dysplasia, (2) included all age groups, and (3) evaluated the family history effects for IBD or CRC. The total number of IBD patients and IBD patients with a family history of CRC and the total number of CRC patients and CRC patients with a family history of IBD were reviewed. The pooled prevalence of diseases was also estimated according to degree of relatives and geographical area. Random-effects models were used for estimating pooled prevalence.

**Results:**

A total of 27 studies were included with 26,576 IBD and 9,181 CRC or dysplasia patients. Eligible studies included 13 case-control, 10 cohort, and 4 cross-sectional types. The pooled prevalence of a family history of CRC among patients with IBD was 6% (95% CI: 4-9%). The pooled prevalence for first- and second-degree relatives (11%, 95% CI: 0-37%) was more than that for the other relative subgroups of relatedness degree. The prevalence in the American regions (8% (95% CI: 5-13%)) was higher than that in the others. The pooled prevalence for a family history of IBD among CRC or dysplasia patients was 11% (95% CI: 6-16%). The pooled prevalence for first-degree relatives (13% (95% CI: 3-28%) was higher than that for the other relative subgroups of relatedness degree; it was also greater in American countries (15%, 95% CI: 8-23%).

**Conclusion:**

This study emphasizes the relationship between a family history of IBD and CRC development. Additionally, there was notable prevalence for a family history of CRC among IBD patients. American countries and first-degree relatives were identified to have a higher prevalence for both disease processes.

## 1. Introduction

Colorectal cancer (CRC) is one of the most common cancers worldwide [1]. Familial studies have demonstrated that having a family history of CRC may increase an individual's risk of developing CRC and that this risk in individuals with a first-degree relative with CRC is more than 2-folds greater [2, 3]. One of the most important risk factors of CRC is inflammatory bowel disease (IBD). IBD is an immune-mediated gastrointestinal disorder that is identified with subtypes of Crohn's disease (CD) and ulcerative colitis (UC) [4]. Both CD and UC patients are at risk of CRC development [5]. Like CRC, family history is one of the strongest risk factors for development of IBD [6].

It has been shown that CRC is a relatively common and life-threatening consequence of IBD, especially UC. This is likely secondary to proneoplastic effects of chronic intestinal inflammation. Duration, extent and severity of IBD, the existence of inflammatory pseudopolyps, presence of primary sclerosing cholangitis, and a family history of CRC are the main risk factors of IBD-related CRC [7]. A family history of CRC independently increases CRC risk two- to threefolds in patients with UC (OR: 3.7, 95% CI: 1.0–13.2) [8].

There are common factors inducing the development of IBD and CRC, such as the variations in gut microbiota and in the interleukin pathways and tumour necrosis factor, as well as also age, race, genetics, family history, diet composition, obesity, and vitamin and mineral levels [9]. Moreover, it is shown that IBD-related CRC patients are younger and have high prevalence of multiple cancerous lesions [10]. This suggests that in addition to inflammation, other factors may be involved in the pathogenesis of IBD-related CRC.

To determine a quantitative data for the prevalence of a family history of CRC or IBD, there are only a few comprehensive studies. In a previous meta-analysis, the prevalence of CRC in patients with UC has been estimated at 3.7%, across the world [11]. In a study by Shi et al., the prevalence of a family history of IBD among groups of Caucasians, Asians, Blacks, and Hispanics has been estimated at 12%, 0.04%, 0.07%, and 0.13%, respectively [12]. In the other study by Childers et al., it was revealed that the prevalence of a family history of IBD among patients with UC is 12% (range: 0-39%) [13].

Despite CRC and IBD being closely interrelated, the relation between a family history of each disease and the risk of developing the other still has not been quantified. To address this gap, we performed a systematic review and meta-analysis for estimation of the prevalence of a family history of CRC among patients with IBD as well as the prevalence of a family history of IBD among patients with CRC.

## 2. Methods

### 2.1. Search Strategy

Our electronic search was limited to the English language, and it was conducted in PubMed, Scopus, Embase, Web of Science, and Google Scholar by using the following keywords: (“inflammatory bowel disease” or “ulcerative colitis” or “crohn's disease”) and (“colorectal cancer” or “colon and rectum cancer” or “dysplasia or neoplasia”) and (“family history” or “relative” or “familial”). Published studies up to December 2020 were considered, and references of individual studies were searched to find other eligible studies. The Preferred Reporting Items for Systematic review and Meta-Analysis (PRISMA) guideline was used for reporting this study [14].

### 2.2. Inclusion and Exclusion Criteria

The authors reviewed titles and abstracts of original full-text articles performed on each of the IBD or CRC patients. The inclusion criteria were as follows: (1) studies that evaluated either IBD or CRC and dysplasia, (2) included all age groups, and (3) evaluated the family history effects for IBD or CRC. The exclusion criteria were as follows: (1) studies with an unknown number of patients with a family history for IBD or CRC and (2) conducted on animals (mice). The authors excluded all reviews or conference abstracts and non-English publications. For quality control of studies, the authors used the Newcastle-Ottawa Scale (NOS) and the high and moderate quality articles considered as eligible [15]. The disagreements among the authors on the choice of the eligible studies were discussed, and finally, any disagreement was evaluated by the senior investigator.

### 2.3. Data Extraction

For each selected study, the authors extracted the following information: name of the first author, year of publication, country of publication, total sample size, study design, total number of IBD patients and IBD patients with family history of CRC, total number of CRC patients and CRC patients with family history of IBD, and degree of relatives included.

### 2.4. Outcome of Interest

The main outcomes of this meta-analysis were the prevalence of a family history of IBD in CRC as well as the prevalence of a family history of CRC in IBD.

### 2.5. Statistical Analysis

All analyses were done using Stata 14 software, and 0.05 was considered as the statistical significance level. For each outcome of interest, the corresponding proportion was calculated via the extracted data from each eligible study. Pooled prevalence with 95% confidence interval (CI) was estimated using the random-effects model wherever the prevalence has been reported. In the process of prevalence merging, the outcomes with zero event were adjusted using the “Freeman-Tukey double arcsine” transformation in the “metaprop” procedure [16]. The heterogeneity was evaluated by using the Cochran's *Q* test and *I*^2^ statistic and *P* value. We performed stratified analysis for items that may cause heterogeneity. Publication bias was examined by using Begg's and Egger's tests [17] and also funnel plot [18].

## 3. Results

### 3.1. Process of Study Selection

**Figure 1:**
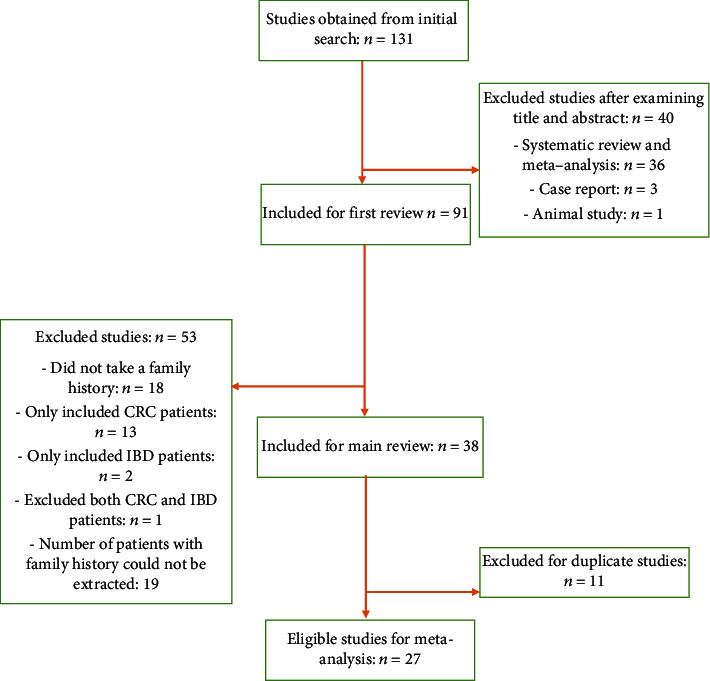
Flow chart for the process of study selection.

After a comprehensive search of the databases, 131 studies were obtained. We excluded 40 studies after examining the title and abstract. The number of studies selected for primary evaluation was 91. Then, 53 studies were excluded because they did not meet inclusion criteria or did not report all necessary information. There were 11 republished studies that contained duplicate data samples which were excluded. Finally, 27 studies were considered as eligible and enrolled in the meta-analysis. The details of study selection are presented in Figure 1.

### 3.2. Characteristics of the Eligible Studies

Eligible studies included 13 case-control, 10 cohort, and 4 cross-sectional types [8, 19–44]. The interest disease which was considered in most studies was IBD-related CRC, and the main target was often evaluation of the factor associated with CRC. Some cohort studies such as Brackmann et al. followed up patients with IBD and evaluated the influence of family history of CRC on survival [19]. Other cohort types including Askling et al.'s study followed up UC and CD patients, and they concluded that having both family histories of IBD and CRC increases the risk of CRC [20]. In some of the case-control studies, the main aim was to review supplementation (aminosalicylate and folic acid) effect on the risk of IBD or CRC [21, 22]. The characteristics of the eligible studies are presented in Table 1. The number of IBD and CRC or dysplasia patients represented by 27 eligible studies was 26,576 and 9,181, respectively. All studies except three reported the age at IBD diagnosis. For IBD patients, the mean age at IBD diagnosis was 34.52 ± 7.79 years (range: 25-29), and for IBD patients with CRC or dysplasia, this mean was 35.35 ± 8.65 years (range: 25-57.4).

### 3.3. Family History of CRC among IBD Patients

**Figure 2:**
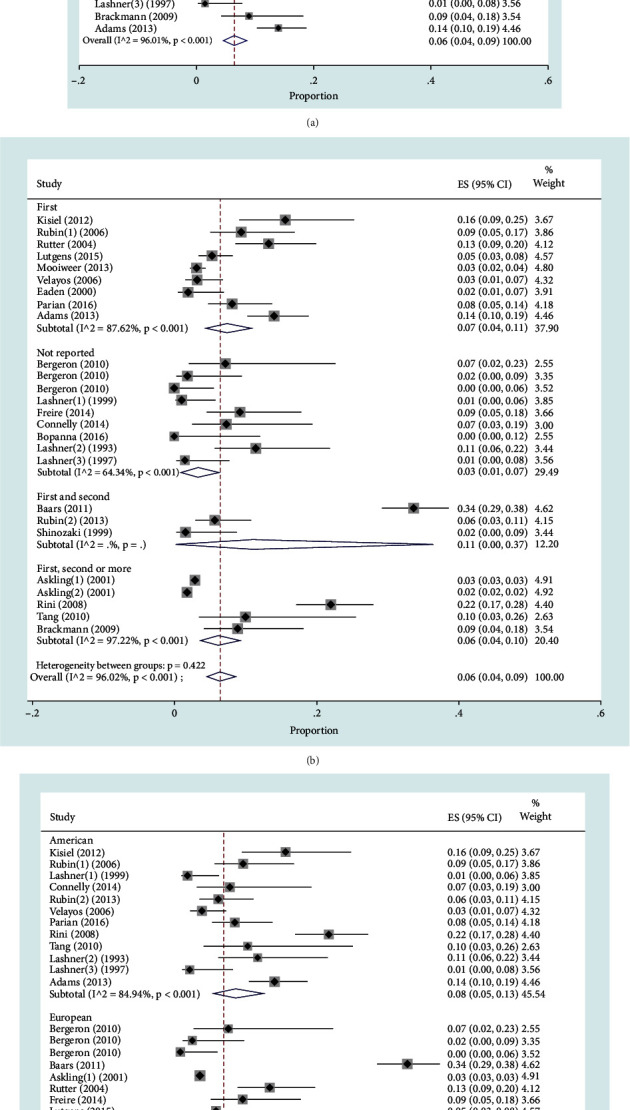
Forest plot for the prevalence of family history of CRC among IBD patients: (a) overall prevalence; (b) prevalence by degree of relative; (c) prevalence by region of study.

There were 26 studies on the family history of CRC, including 25,819 IBD patients. The pooled overall prevalence of a family history of CRC among patients with IBD was 6% (95% CI: 4-9%, *P* < 0.001) with *I*^2^ = 96.01%, *P* < 0.001. The forest plot of the result is presented in Figure 2(a).

#### 3.3.1. Family History of CRC among IBD Patients by Degree of Relative

The degrees of relatives in the extracted studies were reported as first (including 9 studies with 2,357 IBD patients), first and second (including 3 studies with 594 IBD patients), all degrees (including 5 studies with 22,316 IBD patients), and not reported degree (including 5 studies with 492 IBD patients). More studies were conducted on first-degree relatives, while there were studies that did not report any degree of family connection. The pooled prevalence of a family history of CRC among patients with IBD for first- and second-degree relatives (11%, 95% CI: 0-37) was more than any other degree of relatedness (Figure 2(b)).

#### 3.3.2. Family History of CRC among IBD Patients by Region of Study

The studies were conducted in the regions of the Americas (including the USA with 1,419 IBD patients), Europe (including Sweden, France, Netherlands, England, Portugal, and Norway with 24,279 IBD patients), and Asia (including Japan and India with 89 IBD patients). The pooled prevalence of a family history of CRC among patients with IBD in the American regions (8% (95% CI: 5-13%)) was higher than that in the others (Figure 2(c)).

### 3.4. Family History of IBD among Patients with Dysplasia or CRC

**Figure 3:**
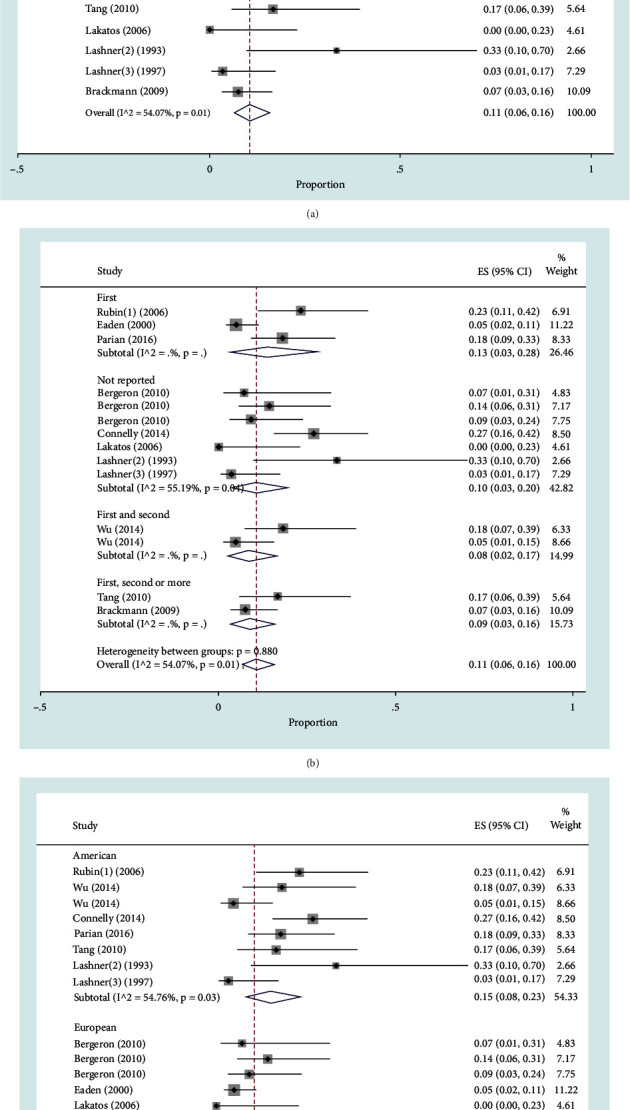
Forest plot for the prevalence of family history of IBD among dysplasia or CRC patients: (a) overall prevalence; (b) prevalence by degree of relative; (c) prevalence by region of study.

The number of studies concerning family history of IBD for CRC patients was 10, including 481 patients with dysplasia or CRC. The pooled prevalence for a family history of IBD among CRC or dysplasia patients was 11% (95% CI: 6-16%, *P* < 0.001) with *I*^2^ = 54.57%, *P* = 0.01. The forest plot is shown in Figure 3(a).

#### 3.4.1. Family History of IBD among Patients with Dysplasia or CRC by Degree of Relative

First degree (including 167 patients with dysplasia or CRC), first and second degrees (including 65 patients with dysplasia or CRC), and first, second, and more degrees (including 85 patients with dysplasia or CRC) were among the reported degree of relatives in the eligible studies. Also, some studies did not report the degree (including 164 patients). The pooled prevalence in the first degree (13% (95% CI: 3-28%) was higher than that in the other groups (Figure 3(b)).

#### 3.4.2. Family History of IBD among Patients with Dysplasia or CRC by Region of Study

The regions of the Americas (including the USA, with 224 dysplasia or CRC patients) and Europe (including France, Norway, Hungary, and Sweden with 257 dysplasia or CRC patients) were the areas represented in the studies reviewed. The pooled prevalence for American countries (15%, 95% CI: 8-23%) was greater than that for the European region (Figure 3(c)).

### 3.5. Evaluation of Publication Bias

**Figure 4:**
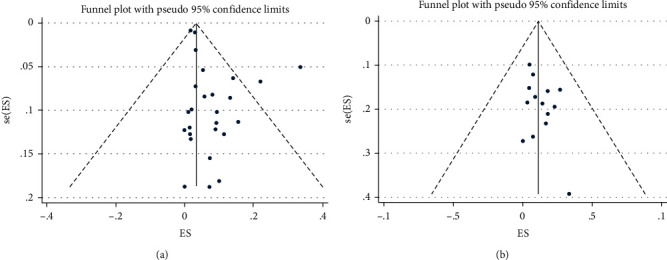
Funnel plot for assessing publication bias in the eligible studies: (a) studies provided data on family history of CRC; (b) studies provided data on family history of IBD.

The results of Egger's (*P* = 0.08) and Begg's (*P* = 0.48) tests revealed that there is no publication bias among the studies from which the prevalence of family history of CRC was. Also, for family history of IBD, the tests of Egger (*P* = 0.78) and Begg (*P* = 0.32) showed similar results. Additionally, the funnel plots showed evidence of an approximate symmetry (Figure 4).

## 4. Discussion

The present study is the first meta-analysis that estimates the prevalence of a family history of CRC among patients with IBD. Interestingly, we found that among IBD patients, the prevalence of a family history of CRC was 6% (95% CI: 4-9). Additionally, the pooled prevalence of a family history of IBD among patients with CRC or dysplasia was estimated to be 11% (95% CI: 6-16). The reason for the greater latter prevalence may be that previous cohort studies proved that the history of IBD is a factor associated with CRC and the probability of developing CRC for IBD patients in the future is 2-folds higher compared with that for others [45–47]. It is important to note that IBD patients with longer duration and extensive disease and patients with diagnosis at young age are at higher risk of CRC [48]. Among IBD patients, also, the prevalence of a family history of CRC may be notable and the present study confirmed this.

In a recent meta-analysis on the influence of ethnicity in IBD prevalence by Shi et al., the mean age of IBD diagnosis was reported as 30 years [12]. This mean age in other epidemiological studies has varied, with reports of 32.7 [49], 38.46 [50], and 54.1 [51] years of age. These differences may be due to differences in access to health care center for diagnosis and overall awareness about IBD. In our study, we observed that the mean age of IBD diagnosis for IBD patients was close to 34.52 (range: 25-29), and for IBD patients with CRC, this was slightly later (35.35, range: 25-57.4). This may mean that at a later mean age of diagnosis, patients with IBD are at higher risk for CRC than young people [52, 53].

In our study, the authors reported the pooled prevalence of family history of CRC among patients with IBD as well as a pooled prevalence of a family history of IBD among patients with CRC or dysplasia, according to the degree of relatedness. The result demonstrated that the prevalence of family history for first- or first- and second-degree relationships is greater than that for other degrees. Previous studies revealed that the risk of gastrointestinal cancers for individuals with affected family members, especially for first-degree family members, is high and our result is in line with this point [20, 54].

Considering the geographical aspect, the present meta-analysis revealed that the pooled prevalence of family history of either IBD or CRC among Americans was more than that among European and Asian countries. Perhaps the global cancer statistics are helpful for finding the reason: the incidence of CRC is more common in more highly developed countries. The CRC incidence rate in Europe and Northern America is highest in comparison with that in other regions. The reason is that the prevalence of CRC risk factors including obesity and unhealthy diet in these regions is high [55, 56]. Additionally, global comparison for the prevalence of IBD has shown the highest prevalence in European and American areas and the prevalence has remained higher up until 2018 [57].

For IBD, diagnosis and management are complex and utilize clinical presentation, biomarkers, and pathology. Patient manifestation of symptoms may be due to genetic, environmental factors, and possibly molecular mechanisms within the gut microbiota patterns. All of these areas may be targets for personalized IBD treatment. This tailored approach is important for early diagnosis and treatment in the IBD management [58, 59]. Early IBD diagnosis and successful treatment may result in decreased rates or prevention of CRC.

Previous studies observed significant heterogeneity among the results of studies. The heterogeneity is a phenomenon that usually is seen in the meta-analyses of proportion. Instead, we performed a subgroup analysis to create some more homogeneous groups of studies. But the subgroup analysis is performed by dividing studies into stratum, and this may not be useful in all cases. Generally, the more likely cause of heterogeneity, in addition to measurement errors, may be due to the way of constructing the study, including methods as well as differences in the span of the definitions [60].

There are some limitations in the present meta-analysis. First, there is overall a lack of determining the number of patients with affected family member for subtypes of UC or CD in the eligible studies. This limitation caused the authors to not take the two main types of pooled prevalence for each subtype. Second, some eligible studies did not report the degree of relatedness for affected family members. Also, for first-degree relatives, the type of relative (parent or sibling) was not mentioned. With more complete data, the results could be expanded. Further genetic studies are needed to determine the number of subjects with family affected member for both IBD and CRC in details of IBD subtypes, as well as sex in each type.

The present study emphasized the importance of a family history of IBD (or CRC) in the possibility of the CRC onset (or IBD). The advancement of CRC in non-IBD patients, with family history of IBD, leads us to look up further probable factors that may be common among both diseases. Gut microbiota, interleukin, and tumour necrosis factor pathways, race, genetics, family history, and diets are important factors that should be considered in the future studies [9]. The prevalence of a family history of CRC among IBD patients in American and European countries and for first-degree relatives is higher. There is a similar pattern for the prevalence of a family history of IBD among dysplasia or CRC patients. Thus, knowing the prevalence of a family history component for an at-risk population may be helpful in patient's care and managing both CRC and IBD.

## Figures and Tables

**Table 1:** Characteristics of studies included in the meta-analysis.

Study	Year	Region	Sample size	Study type	Mean age at diagnosis of IBD (year)	No. of patients with dysplasia and CRC	No. of IBD patients; UC, CD	Family history of CRC	Family history of IBD	Relevant degree	Proportions^∗^	
Kisiel et al. [23]	2012	USA	77	Cohort: followed 77 IBD patients	47.6 (14.2–83.2)	31	77 UC patients under polypectomy	12	—	First	$p_{1}=\frac{12}{77}$	
Bergeron et al. [24]	2010	France	855	Cohort: followed 855 IBD patients	33 (19-47)	14 with AL-LGD^a^	28	2	1	—	$p_{1}=\frac{2}{28};p_{2}=\frac{1}{14}$	
						28 with CR-LGD^b^	56	1	4	—	$p_{1}=\frac{1}{56};p_{2}=\frac{4}{28}$	
						33 with advanced neoplasia	66	0	3	—	$p_{1}=\frac{0}{66};p_{2}=\frac{3}{33}$	
Baars et al. [25]	2011	Netherlands	565	Case- (CRC with IBD) control (IBD)	33 for cases, 31 for control	173 (UC:113; CD: 58; unclassified: 2)	392 (UC: 175; CD: 207; unclassified: 10)	132	—	12 first, 11 second, 109 unknown	$p_{1}=\frac{132}{392}$	
Rubin et al. [26].	2006	USA	125	Case- (UC with developed dysplasia or CRC) control (UC without neoplasia)	29.5 for cases, 30.5 for control	26	96	9	6	First	$p_{1}=\frac{9}{96};p_{2}=\frac{6}{26}$	
Lashner et al. [27]	1999	USA	95	Cohort: followed 95 UC patients	26.5 for p53-positive, 29.5 for p53-negative	36	95	1	3	—	$p_{1}=\frac{1}{95}$	
Askling et al. [8]	2001	Sweden	8810	Cohort: followed 8810 CD patients	—	143	8810	256	—	First, second, or more	$p_{1}=\frac{256}{8810}$	
Rutter et al. [29]	2004	England	204	Case- (CRC neoplasia) control (UC)	33 for cases, 33 for controls	68	136	18	—	First	$p_{1}=\frac{18}{136}$	
Wu et al. [28]	2014	USA	44	Cohort: followed 44 UC patients	29.3	22 with LGD	44	—	2	First or second	$p_{2}=\frac{2}{22}$
					28.2	43 with adenocarcinoma and pouch dysplasia		—	4		$p_{2}=\frac{4}{44}$
Freire et al. [30]	2014	Portugal	76	Cross-sectional in UC	33.3	—	76	7	—	—	$p_{1}=\frac{7}{76}$
Connelly et al. [31]	2014	USA	82	Case- (UC with dysplasia/CRC) control (UC without dysplasia/CRC)	34.49	41	41	3	11	—	$p_{1}=\frac{3}{41};p_{2}=\frac{11}{41}$
Lutgens et al. [32]	2015	Netherlands	530	Case- (IBD-CRC) control (IBD)	27.5 for cases, 25.5 for control	188	342	18	—	First	$p_{1}=\frac{18}{342}$
Rubin et al. [33]	2013	USA	200	Case- (CRC and UC) control (UC	47.1	59	141	8	—	First or second	$p_{1}=\frac{8}{141}$
Shinozaki et al. [34]	1999	Japan	77	Case- (UC with neoplasia) control (UC)	34.3 for cases, 28.4 for control	16	61	1	—	First or second	$p_{1}=\frac{1}{61}$
Mooiweer et al. [35]	2013	Netherlands	1018	Cohort: followed 1018 IBD patients	47.7	11	1018	32	—	First	$p_{1}=\frac{32}{1018}$
Velayos et al. [36]	2006	USA	376	Case- (UC with CRC) control (UC)	25 for cases, 27 for control	188	188	6	—	First	$p_{1}=\frac{6}{188}$
Askling et al. [20]	2001	Sweden	31093	Cohort: followed 31093 IBD patients	37.65	560	13186	234	—	First, second or more	$p_{1}=\frac{234}{13186}$
Eaden et al. [37]	2000	Sweden	204	Case- (UC, CRC) control (UC)	57.4 at diagnosis of CRC	102	102	2	5	First	$p_{1}=\frac{2}{102};p_{2}=\frac{5}{102}$
Bopanna et al. [38]	2016	India	28	Cross-sectional in UC	28.89	—	28	0	—	—	$p_{1}=\frac{0}{28}$
Parian et al. [39]	2016	USA	187	Cross-sectional in UC	36.7 for UC with dysplasia, 29.7 for UC without dysplasia	39	148	12	7	First	$p_{1}=\frac{12}{148};p_{2}=\frac{7}{39}$
Rini et al. [40]	2008	USA	223	Cross-sectional in IBD	43.94	—	223: 136 UC, 55 CD, 32 unknown	15 in first degree; 34 in second or more	—	First, second, or more	$p_{1}=\frac{49}{223}$
Tang et al. [21]	2010	USA	48	Case- (IBD with CRC) control (IBD)	36.6 for cases, 34.7 for control	18	30	3	3	First, second, or more	$p_{1}=\frac{3}{30};p_{2}=\frac{3}{18}$
Lakatos et al. [41]	2006	Hungary	723	Cohort: followed 723 UC	49	13	723	—	0	—	$p_{2}=\frac{0}{13}$
Lashner [42]	1993	USA	67	Case- (UC with dysplasia and CRC) control (UC)	—	6	61	7	2	—	$p_{1}=\frac{7}{61};p_{2}=\frac{2}{6}$
Derikx et al. [43]	2014	Netherlands	124	Case- (IBD with carcinoma and dysplasia) control (IBD)	25.7 for cases, 25.7 for control	25	99	0	—	—	$p_{1}=\frac{0}{99}$
Lashner et al. [22]	1997	USA	98	Case- (UC with CRC or dysplasia) control (UC without CRC or dysplasia)	25.8 for CRC or dysplasia, 29.5 for no dysplasia	29	69	1	1	—	$p_{1}=\frac{1}{69};p_{2}=\frac{1}{29}$
Brackmann et al. [19]	2009	Norway	67	Cohort: followed 67 IBD with CRC	25	67	67	6	5	First, second or more	$p_{1}=\frac{6}{67};p_{2}=\frac{5}{67}$	
Adams et al. [44]	2013	USA	7202	Cohort: followed 7202 CRC cases	—	7202	250	35	—	First	$p_{1}=\frac{35}{250}$	

^∗^ *p*_1_ = patient with family history of CRC/patient with IBD; *p*_2_ = patient with family history of IBD/patient with dysplasia or CRC; ^a^adenoma-like low-grade dysplasia; ^b^colitis-related low-grade dysplasia.

## Data Availability

There is no raw data associated with this article.
